# Material Flow Analysis in Indentation by Two-Dimensional Digital Image Correlation and Finite Elements Method

**DOI:** 10.3390/ma10060674

**Published:** 2017-06-21

**Authors:** Carolina Bermudo, Lorenzo Sevilla, Germán Castillo López

**Affiliations:** Department of Civil, Material and Manufacturing Engineering. EII, University of Malaga, 29071 Malaga, Spain; lsevilla@uma.es (L.S.); gcastillo@uma.es (G.C.L.)

**Keywords:** incremental forming, indentation, Digital Image Correlation, Finite Elements Method, experimental methodology

## Abstract

The present work shows the material flow analysis in indentation by the numerical two dimensional Finite Elements (FEM) method and the experimental two-dimensional Digital Image Correlation (DIC) method. To achieve deep indentation without cracking, a ductile material, 99% tin, is used. The results obtained from the DIC technique depend predominantly on the pattern conferred to the samples. Due to the absence of a natural pattern, black and white spray painting is used for greater contrast. The stress-strain curve of the material has been obtained and introduced in the Finite Element simulation code used, DEFORM™, allowing for accurate simulations. Two different 2D models have been used: a plain strain model to obtain the load curve and a plain stress model to evaluate the strain maps on the workpiece surface. The indentation displacement load curve has been compared between the FEM and the experimental results, showing a good correlation. Additionally, the strain maps obtained from the material surface with FEM and DIC are compared in order to validate the numerical model. The Von Mises strain results between both of them present a 10–20% difference. The results show that FEM is a good tool for simulating indentation processes, allowing for the evaluation of the maximum forces and deformations involved in the forming process. Additionally, the non-contact DIC technique shows its potential by measuring the superficial strain maps, validating the FEM results.

## 1. Introduction

The indentation process is considered a secondary process due to produced deformations. These deformations are localized, small, and superficial [1,2]. Indentation is generally known as a hardening test.

Nowadays, new complex functional components are demanded with reduced weight and local strength, for example, for gear elements manufacturing. Localized forming operations are an interesting alternative to conventional machining processes [3]. As a manufacturing process, indentation is increasingly adapting to the metalworking industry. New flexible processes are arising and the indentation process implementation is being analysed under different innovative approaches, such as the Incremental Forming Processes (IFP) [4]. The IFP are considered an alternative to traditional plastic forming processes. The final shapes are gradually obtained using dies smaller than the workpieces. These processes modify the material thickness in specific areas, causing permanent plastic deformation and change the material properties as well with repetitive impressions.

Additionally, the incremental techniques can be found in micro forming. Micro-bulk forming produces high quality components with no material waste and is faster than traditional techniques [5]. The aim is to export these bases to general manufacturing.

IFP present important advantages over conventional processes, highlighting the flexibility and lower forces needed [6] that improve the processes. The main challenge of the Sheet-Bulk Metal Forming (SBMF) is the material flow prediction and control. For complex components, “trial and error” is still the most feasible technique [7,8,9,10,11].

Currently, the manufacturing industry requires more reliable and efficient analysis tools. To optimize manufacturing and design these components, the materials’ behaviour laws and consistent simulation tools are needed. This is important for every industry, but for the manufacturing industry it is urgent due to the necessity of knowing the material behaviour within high deformation ranges and, occasionally, near to failure.

Tensile strength trials using strain gauges are the most usual tests. However, this method provides the average deformation values along the strain gauge length, leading to strong discrepancies between the maximum values obtained. Usually, strains and ultimate tensile strengths are larger than the ones obtained with this system [12].

Among the different techniques for the strain measurements, Digital Image Correlation (DIC) has several outstanding advantages [13]:

- It is a non-contact method that eliminates or reduces the interactions with the specimen. This technique can be applied with the samples at high or low temperatures or other extreme conditions without changes in the measurement technique.
- There is no need for complex tools or specimen preparation. Only a charge-coupled device (CCD) camera is needed and, in case the specimen does not count with an appropriate pattern, paint for the specimen surface to create the pattern.
- Natural or white light is used. There is no need of a laser source.
- It provides a wide measurement range and great sensitivity and resolution.

The Finite Elements Method (FEM) is a numerical method widely used for the simulation of materials, components, or structures under different forces. Detecting material deformation, plastic yielding, and damage is always of great importance. On the one hand, FEM and general numerical simulations are replacing more expensive and complex experiments. Its applications enable simulations of deformation processes with an extensive range of materials. On the other hand, this methodology is as powerful as the mathematical models behind them. Therefore, it is necessary to determine the particular material behaviour law [14]. The FEM software usually includes material databases that consider the material behaviour, deformation, hardening laws, and the strain velocity dependence. Nevertheless, sometimes those models are not accurate enough under extreme conditions, which leads to experimental validations to generate new material models. Additionally, extreme simulation conditions require remeshing techniques to avoid element distortion and numerical errors, and therefore need more computational time [13,15]. FEM is still an expensive approach due to the need of an accurate simulation model.

The goal of the present research is to perform a material flow analysis in an indentation process by FEM and DIC. A single indentation is analysed as the first stage of a SBMF process. In order to be more confident with the approaches implemented, the two methods have been compared. A 99% tin material is used to achieve higher penetrations and avoid crack formation. Regarding indentation, obtaining the full-field displacement during the process improves the understanding of the mechanisms involved in the deformation process. DIC application offers the opportunity to measure displacements and strains on the sample surface in real time, obtaining the whole field with a wide measurement range [16,17].

## 2. Materials and Methods

The study considers two different approaches: Experimental tests and numerical analysis.

Two different experimental tests have been performed: A compression test to obtain the stress-strain law that represents the material behaviour, and indentation tests.

The Digital Image Correlation (DIC) method has been used to measure the sample deformation during indentation tests.

The compression stress-strain curves have been implemented in the FEM model. Once the material behaviour has been experimental and numerically correlated, the indentation test has been modelled. The resulting indentation forces-displacement curves are compared to validate the numerical model with the experimental procedure. After the validation, the von Mises strain distribution obtained from the FEM and DIC methods can be compared.

### 2.1. Digital Image Correlation

**Figure 1:**
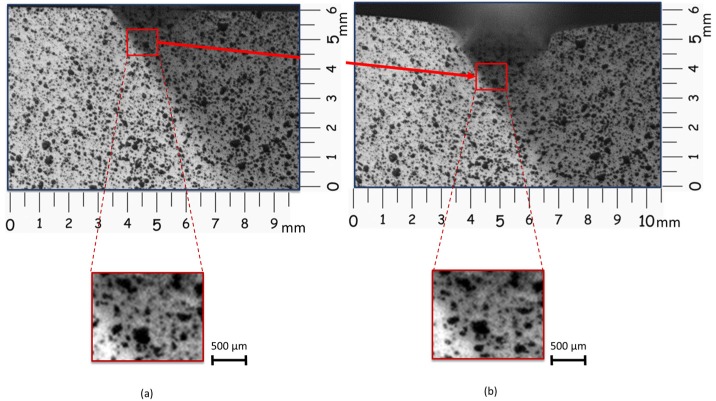
Subset before (**a**) and after (**b**) deformation.

The 2D DIC technique is based on the identification and comparison of a zone on the surface of the workpiece or specimen before and after deformation (Figure 1). The image is divided by virtual subsets. This area (subset) has a unique light intensity (grey level) that stays the same during the whole deformation process.

Successive comparisons are made in order to evaluate the subset displacement. Correlation algorithms, like the Sum of Squared Differences (SSD) (Equation (1)), locate the subset in the new image. Every single subset pixel is associated with a number according to its grey level (100 for white and 0 for black), as Figure 1 shows.

(1) $$C\left(x,y,u,v\right)=\overset{\frac{n}{2}}{\underset{i,j=\frac{-n}{2}}{\sum }}{\left(I\left(x+i,y+j\right)-\Gamma \left(x+u+i,y+v+j\right)\right)}^{2}$$

where, *C*(*x*, *y*, *u*, *v*): The value of the correlation function for a given pixel in the position (*x*, *y*) that undergoes a horizontal (*u*) and vertical (*v*) displacement (reference image); *n*: the subset size; *I*(*x* + *i*, *y* + *j*): The value associated to the pixel in the position (*x* + *i*, *y* + *j*) (reference image); Γ(*x* + *u* + *i*, *y* + *v* + *j*): The value associated to the pixel in the position (*x* + *u* + *i*, *y* + *v* + *j*) (deformed image).

The lowest *C* value offers the best correlation possible, giving the new position (*x*, *y*) of the pixel in the image after deformation, as well as the horizontal and vertical displacements (*u*, *v*) [18].

**Figure 2:**
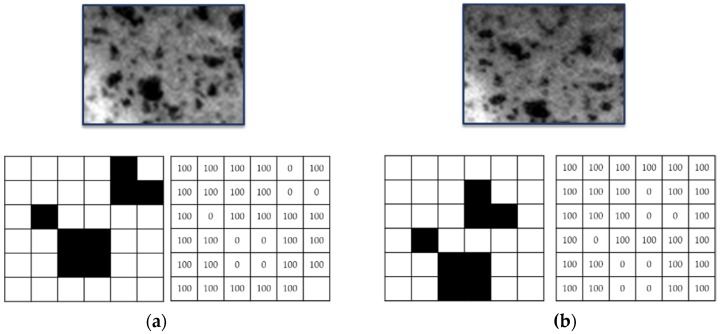
Subset and grey levels before (**a**) and after (**b**) deformation.

However, it is necessary that the analysed specimen shows an adequate pattern. Commonly, this pattern consists of a random mottling or speckle that allows the recognition of the position of every single point before and after deformation. Frequently, the specimen under study presents a natural pattern but it is not uncommon to use other techniques, like spray paint or electrospray, to create these random patterns. Figure 1 and Figure 2 show a pattern made with spray paint.

Once the displacement vectors of each pixel is obtained, it is possible to interpolate any point with the interpolation equations (Equations (2) and (3)):

(2) $$u\left(x,\;y\right)=u_{0}+u_{x}x+u_{y}y$$

(3) $$v\left(x,\;y\right)=v_{0}+v_{x}x+v_{y}y$$

where (*x*, *y*) are the desired point coordinates and (*u*, *v*) is the displacement.

Considering that between two consecutives frames the hypothesis of small strains is applicable, the Cauchy-Almansi tensor can be applied to obtain the strain field (Equation (4)):

(4) $$\varepsilon =\left(\begin{array}{cc} \varepsilon_{xx} & \varepsilon_{yx}\\ \varepsilon_{xy} & \varepsilon_{yy} \end{array}\right)=\left(\begin{array}{cc} u{,}_{x} & \frac{1}{2}\left(u{,}_{y}+v{,}_{x}\right)\\ \frac{1}{2}\left(u{,}_{y}+v{,}_{x}\right) & v{,}_{y} \end{array}\right)$$

where, *ε_xx_* and *ε_yy_* are the longitudinal strains in the *x* and *y* directions, respectively; *ε_xy_* is the angular strain; *u,_x_*, *u,_y_*, *v,_x_*, and *v,_y_* are partial derivatives of the displacements (*u*, *v*).

### 2.2. Materials and Specimens

The material selected to accomplish the deep indentations is 99% tin (NB1101003), obtained from 6 × 10 × 70 mm^3^ bars with a 232–247 °C melting temperature (Table 1). A green sand casting process has been used to produce a 60 × 60 × 70 mm^3^ ingot. All the tests specimens have been machined from that tin ingot to avoid different behaviours due to cooling alterations inside the material.

**Figure 3:**
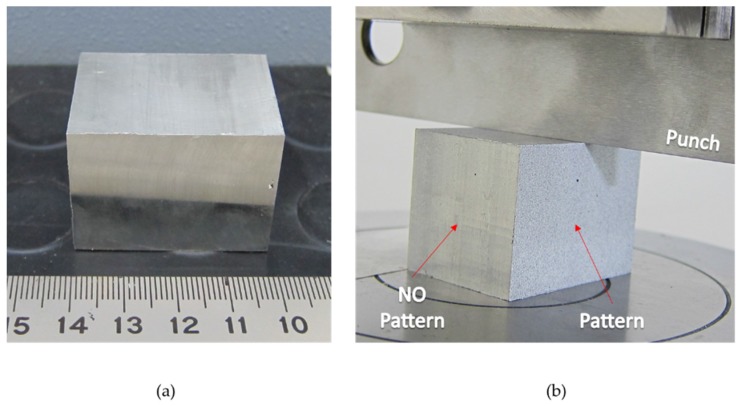
Specimen without pattern (**a**) and with pattern applied (**b**).

Two kinds of specimens are used for the compression and the indentation tests. The ASTM E9 standard has been followed for the compression tests. The dimensions of the specimens are 15 × 15 × 30 mm^3^. The length is established to allow for enough axial deformation without buckling. For the indentation tests, five 99% tin specimens of 40 × 30 × 30 mm^3^ have been prepared to study the material flow under a single indentation (Figure 3). In order to work in plane strain conditions, the specimen depth must be 6 to 10 times the deformation surface width [19].

In this case study, the punch is 3 mm wide, so the specimen depth is set to be 10 times bigger, 30 mm.

**Figure 4:**
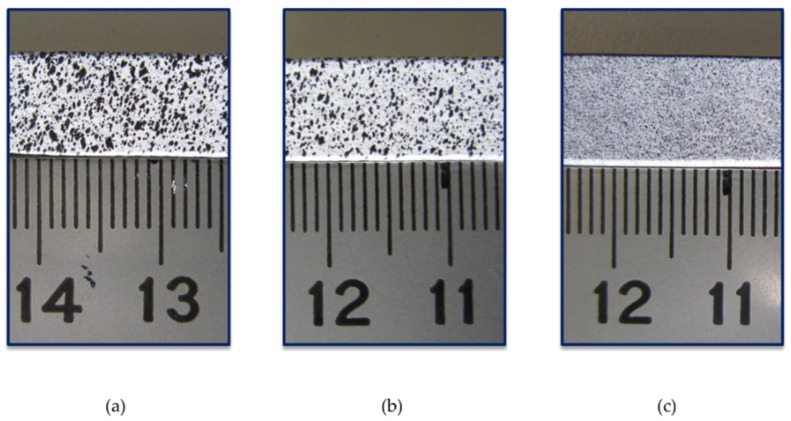
Evolution of the specimen pattern. First attempts (**a**,**b**) and final pattern (**c**).

All workpieces need to be precisely painted to obtain an adequate pattern (Figure 4). It is important to spray the parts at a large distance to prevent the first thicker drops from falling on the surface. With finer patterns, more accuracy can be obtained. First, a white coat is sprayed at a 40–45 cm distance, generating a thin layer. Several layers can be sprayed if necessary. After the white coat dries, a black mottling is sprayed at 100 cm so that the large droplets fall before reaching the specimen (Figure 3b and Figure 4c). Two hours later, the specimens can be tested. It is also important not to let the painting dry completely to prevent paint cracking during indentation.

### 2.3. Experimental Tests

The compression tests were carried out in a universal tension-compression machine Servosis ME 405, equipped with a 20 kN load cell. The test speed was set to 5 mm/min, equal to the indentation test’s speed, to minimize the strain-rate influence. Due to the elasto-plastic material behaviour, it is not necessary to use the DIC technique to evaluate the samples’ deformations.

**Figure 5:**
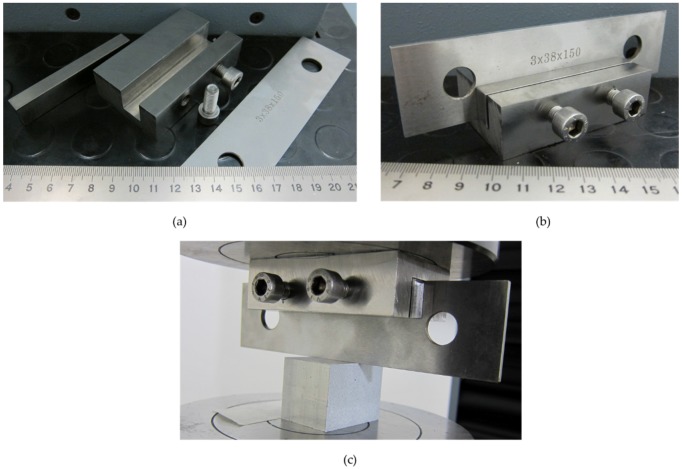
Restraining tool disassembled (**a**), assembled (**b**), and pre-load before test (**c**).

For the indentation tests, a restraining tool was designed to complete the indentation process, avoiding punch inclination (Figure 5). Due to the narrow surface of the punch (3 mm) in contact with the specimen, an inclination of the punch was observed in the first samples tested. Therefore, the use of a restraining tool was necessary to prevent a non-symmetrical deformation. This tool was designed with a lateral compression force which stabilizes the punch, applying the compression with two fixed points on an in-between element. This in-between element homogeneously sets the punch and prevents its lateral displacement. Additionally, the restraining system does not affect the indentation results. A punch of steel AISI 304 is used.

To obtain the image correlation, an Allied digital camera Stingray EEE 1394b of 5 megapixels (Sony, UK) was used, with a cell size of 3.45 µm × 3.45 µm. The camera was equipped with a Pentax C7528-M lens. This lens is specially designed for image processing applications. It is purposely designed to maximise the picture performance at short distances with a 75 mm focal length. With these characteristics, the pixel size is 30 µm.

**Figure 6:**
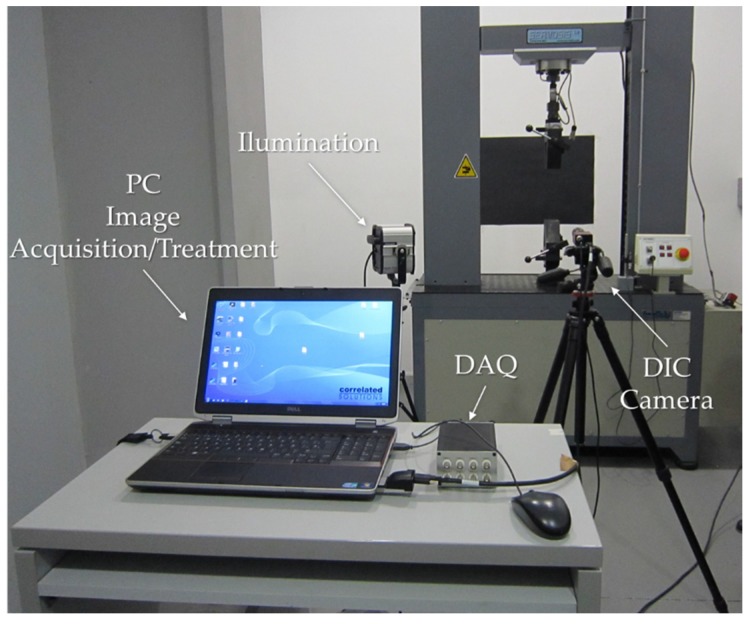
Image acquisition system.

For the illumination of the set, a Hedler spotlight DX 15 (metal 150 W Halide lamp, Hedler Systemlicht, Runkel, Germany) was used. The frame acquisition frequency is 2 Hz and the image acquisition is made with the software VIC SNAP [20] and VIC 2D [21] for treatment after the test is conducted. Figure 6 shows the disposition of the image acquisition system; seen in the foreground is the Data Acquisition system (DAQ) and the computer used to manage the DIC system. Placed in the background is the illumination and the camera through which the images are captured.

Indentation tests were also carried out with the universal tension-compression machine Servosis ME 405 (Servosis Teaching Machines, Madrid, Spain), equipped with a 20 kN load cell. The speed of the indentation process (5 mm/min) is intended to be slow to capture more images and improve the precision of the displacements. With a high capture speed and low indentation speed, more images can be obtained. According to the ISO 6892-1:2010 standard [22], a minimum of five samples have been tested in order to achieve a minimum of a 95% confidence interval.

Materials like tin are strain rate sensitive [23]. For this reason, the behaviour law of the material selected has been obtained at the same speed (5 mm/min) at which the indentation tests are performed. The load forces and displacements of the tool are measured synchronously with the digital image acquisition. Thus, it is possible to know the load-time and displacement-time evolution.

**Figure 7:**
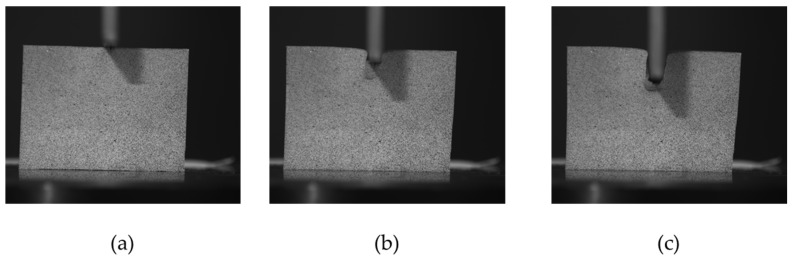
DIC (Digital Image Correlation) analysis. Initial stage, the punch rest on top of the sample (**a**). Middle stage: The punch has penetrated 2–3 mm and the material nose is forming (**b**). Final stage: The indentation process is over and the material nose under the punch is fully created (**c**).

For the DIC analysis, the steps are defined as the number of pixels between correlations. For the image analysis, the subsets and steps are stablished in 45 and 2 pixels, respectively, in order to achieve a confidence below 0.001 pixels. A step size of 2 means that a correlation will be carried out at every other pixel in both the horizontal and vertical directions. Note that the analysis time varies inversely with the square of the step size; i.e., a step size of one takes 25 times longer to analyse than a step size of 5. A low step number leads to a more accurate analysis, but increases the time of analysis. Steps can be on the order from 1 to 50. Figure 7 shows different captures using the DIC method.

### 2.4. Numerical Simulation

**Figure 8:**
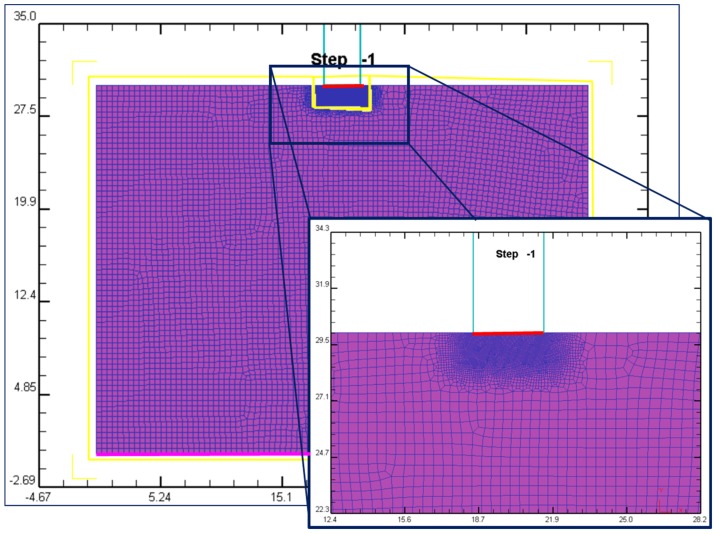
FEM indentation analysis.

The software DEFORM^TM^ 2D (version 8.1, Scientific Forming Technologies Corporation, Columbus, OH, USA) [24] was used for the FEM analysis. This software is specialized in forming process analysis. In order to ensure good results, defining a good mesh distribution is necessary. Near the punch the stress concentration is very high, so a finer mesh is necessary. In this analysis, two different zones have been defined (mesh density windows): One in the contact zone between the punch and the sample, and the other for the rest of the sample. These two zones can be seen in Figure 8.

Based on previous studies, where an indentation process was also simulated with a wider variety of materials [25,26], the optimal mesh is shown in Table 2. A 1/10 relation means that the elements of the second mesh window are 10 times the size of the elements of the first window. This relation guarantees an accurate resolution around the punch, where the main strains are taking place (first window). The rest of the workpiece (second window) is filled with greater elements, providing a shorter computational time resolution.

To avoid the distortions of the big elements, a remesh has been established every 2 steps. Thus, the coarse size element zone becomes larger.

Two different 2D models have been developed; one in plain strain and the second one in plain stress. The two-dimensional plain strain model has been used to obtain the load-penetration curves, since most of the workpiece is near the plain strain conditions. Nevertheless, this model is not appropriate for evaluating strain maps on the surface. The material at the surface is closer to a plane-stress state. Therefore, to achieve this objective, a new 2D plain stress model has been implemented. In both cases the mesh, the type of elements (four node elements), and the boundary conditions are the same.

As boundary conditions, vertical displacements are fixed at the bottom of the sample (pink line, Figure 8). No friction has been considered for the restriction.

On the other hand, a 0.12 shear type friction for cold forming has been defined during the indentation process (red line, Figure 8), between the workpiece and the punch surface. The FEM software used offers different friction values depending on the process simulated, with 0.12 being the appropriate value for cold forming [24]. Notwithstanding, in previous studies [25,26] is it established that the friction force can be neglected due to the total force that the punch needs to achieve to obtain the desired deformation.

**Figure 9:**
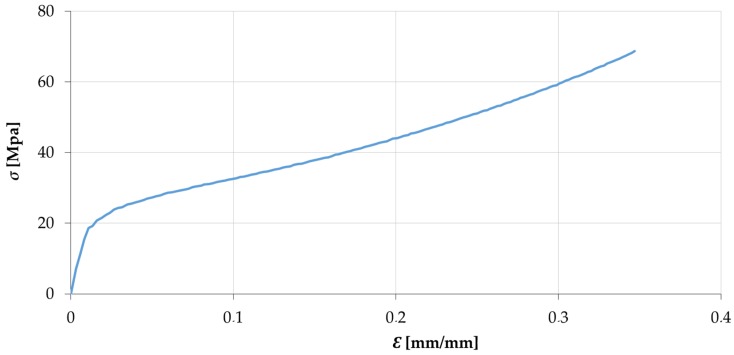
Tin true stress-strain curve obtained from a compression test.

An elasto-plastic isotropic hardening model, based on the von Misses criterion, has been used for the constitutive material model. The material data was previously obtained by the characterization tests and introduced manually in the software database, creating a new material entrance. To manufacture the samples, the tin was melt, cast, and machined. Therefore, the material that is characterized needs to be the same as the indented specimens. Five samples were obtained from these specimens and overcame compression tests in order to obtain the tin stress-strain curves to be implemented in FEM (Figure 9). The true stress and strain are obtained with Equations (5) and (6) [27]:

(5) ε=ln(1+e)

(6) $$\sigma =\sigma_{e}\left(1+e\right)$$

where, e is the engineering strain; σ_e_ is the engineering yield tension; The Newton-Raphson method with 100 steps has been used as the iteration method. For the simulation, the general settings are shown in Table 3.

## 3. Results and Discussion

The aim of the numerical and experimental test correlation is to validate the FEM model. Then, two approaches are used:

- The force-penetration curve along the indentation process.
- Strain maps around the indentation.

In the first approach, load-indentation curves are obtained, one measured directly by the testing machine and the other calculated by FEM. The simulation has been made in 2D plain strain. Thus, the numerical results must be modified to consider the real depth of the workpiece (30 mm).

This load-indentation curve represents the force needed by the punch to achieve the desirable penetration.

**Figure 10:**
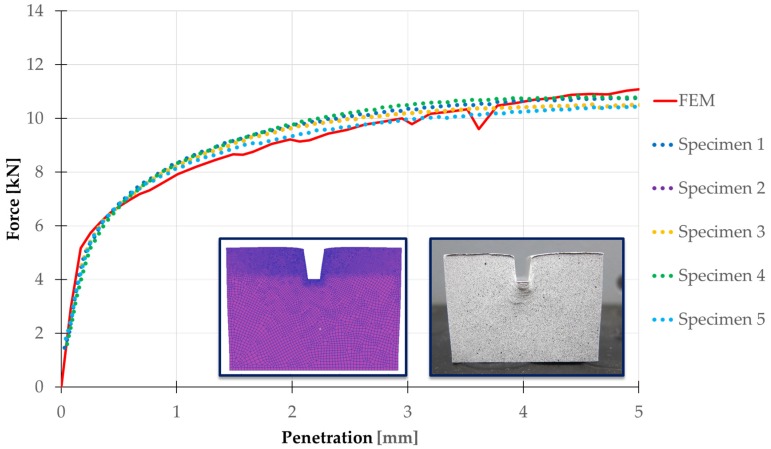
FEM and experimental analysis comparison.

The results obtained by the numerical-experimental correlation (Figure 10) shows that the FEM results agree well with the results obtained from the experimental analysis for the cases studied, which confirms that the FEM analysis is carried out correctly and validates the implemented model. The small discontinuity presented by the curve that corresponds to the simulation is mainly due to the accumulation of errors along the different remesh cycles.

To determine whether the strain distributions around the indentation zone are also well determined by the numerical model, the DIC and FEM results are compared.

**Figure 11:**
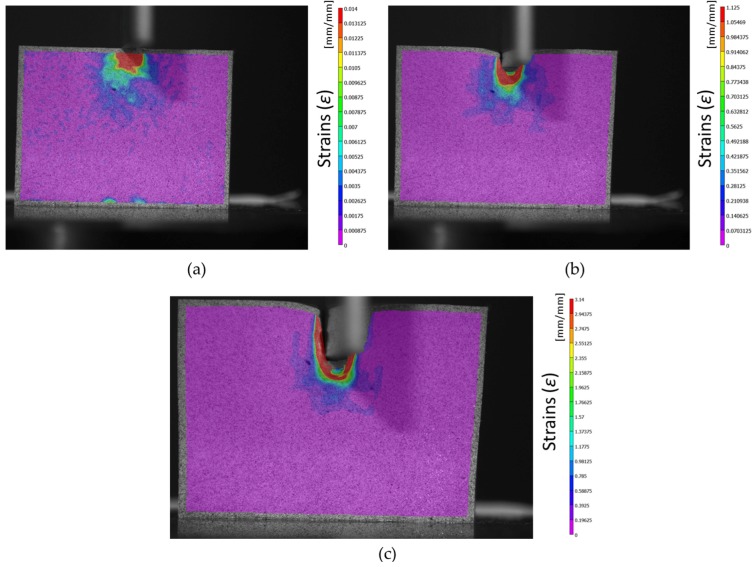
DIC von Mises strain (*ε*) analysis during the indentation process (sample 1). First stage (**a**), middle stage (**b**), and last stage (**c**).

The software used for the DIC analysis, VIC-2D, offers the possibility of analyze the von Mises strains similarly to the FEM analysis code (DEFORM^TM^). In this study, the von Mises strain has been used to represent a single value equivalent to the deformation state at each point. Figure 11 shows the image analysis with DIC for specimen 1, where the von Mises strains are displayedoverlapped. The von Mises strain is the variable through which the DIC-FEM comparison is carried out.

The area of interest (AOI) established by the 2D DIC software is close to the borders of the workpiece during the indentation process, but the information near the borders is not considered, as can be seen in Figure 11.

The gap between the punch and the workpiece that can be seen in Figure 11 is due to the elastic recovery of the material once the indentation process takes place. It can also be seen in the FEM model in Figure 10.

**Figure 12:**
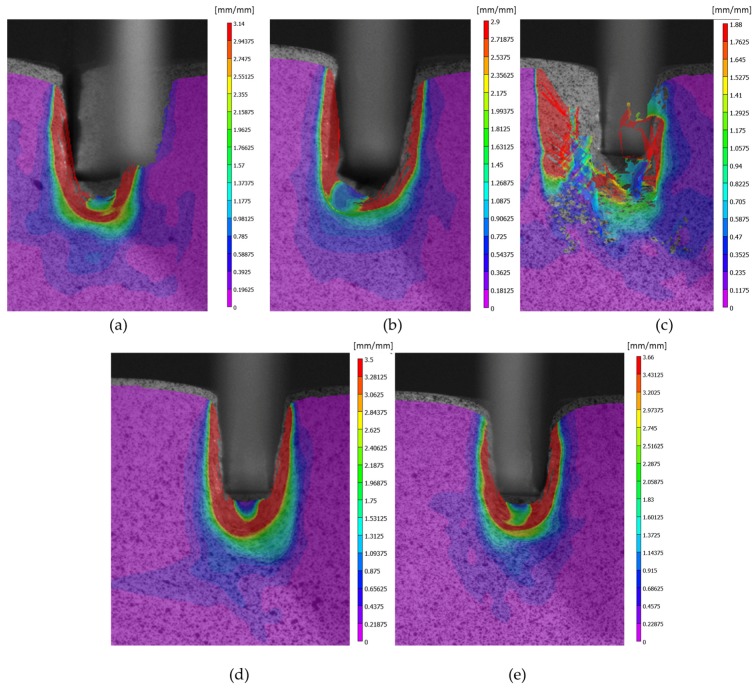
Von Mises strains DIC analysis for specimens 1 (**a**), 2 (**b**), 3 (**c**), 4 (**d**) and 5 (**e**).

Figure 12 shows the image correlation analysis for all of the specimens. It can be appreciated how the program filters/discards from the analysis a zone near the boundaries of the workpiece. This filter is meant to avoid the influence of the non-homogenous boundary values over the mean total values. However, the new boundary zones that are generated while the indentation is taking place (near the punch) are considered in the image analysis. It can be appreciated how the analysis can vary according to the quality of the pattern. The results from specimen 1 (Figure 12a), 4 (Figure 12d), and 5 (Figure 12e) are in good concordance, being results from specimen 4 the ones that show a better correlation . The more appropriate pattern is the one that offers a lower confidence value (*c*), although all of the VIC analyses are below 0.01. VIC 2D calculates the statistical confidence regions using the covariance matrix of the correlation equation. Figure 12c is rejected due to its confidence interval, with Figure 12d being the more representative in this case (*c* = 0.005). The results from specimen 2 (Figure 12b) and 3 (Figure 12c) show defects for deep indentation caused by an overly distorted pattern. This could be due to a coarse pattern. These correlation defects are only present in the latest frames, being the analysis before in good condition. For a single specimen, the total number of frames is 190. Therefore, only part of the analysis is rejected.

**Figure 13:**
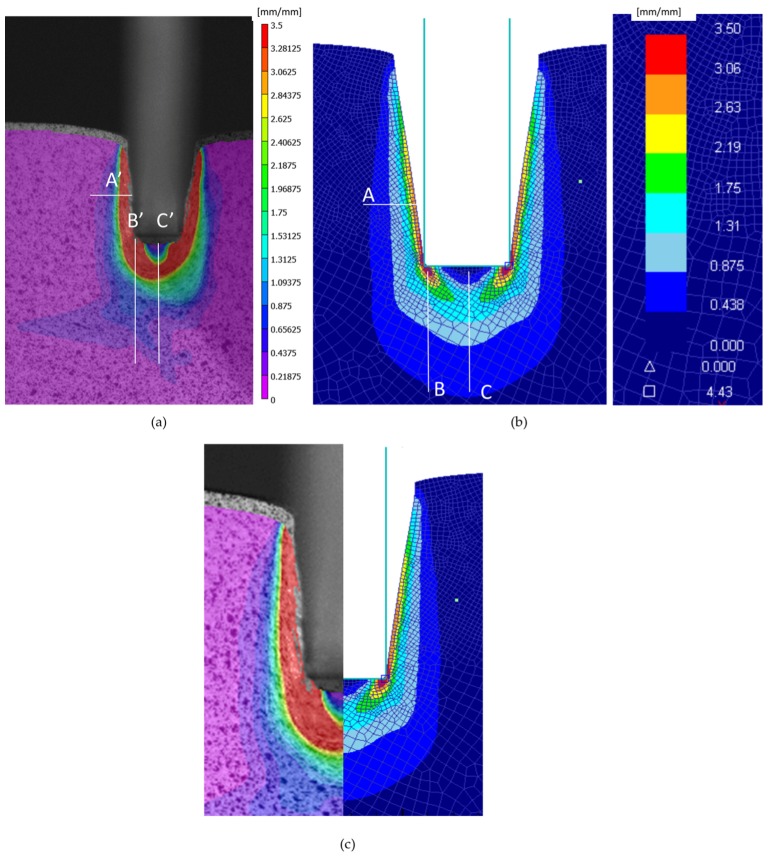
DIC-FEM (2D-Plain Stress) von Mises strain comparison. (**a**) Dic results, (**b**) FEM results and (**c**) comparison.

**Figure 14:**
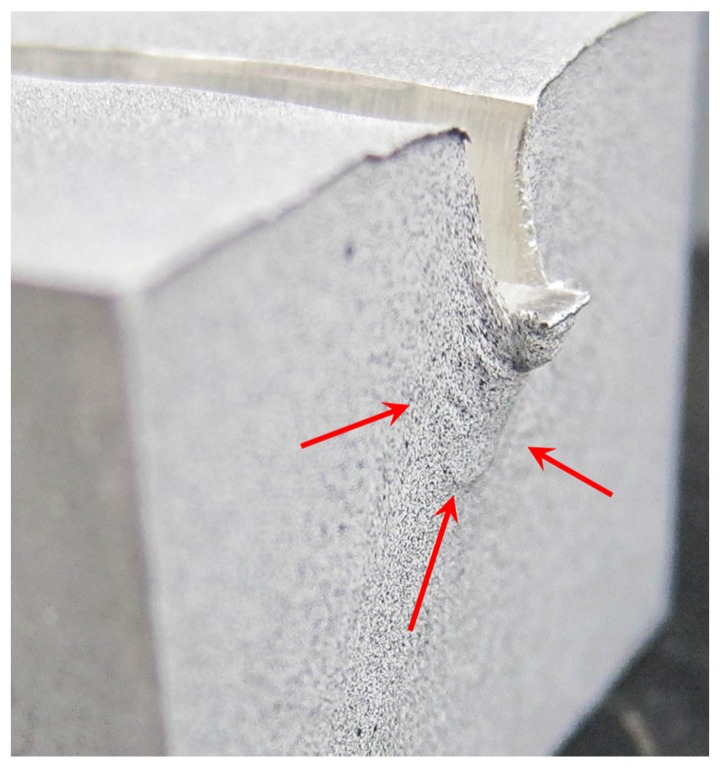
Detail on the sample after the indentation process.

Figure 13 shows a comparison between the FEM two dimensional plain stress model and the DIC model. It can be observed that the DIC resolution is smaller than the numerical results. This can be explained by the pattern used. For a better approximation, the necessary pattern needs to be finer (Figure 4) and focus only on the area of interest, near the punch, taking advantage of the camera resolution. Although the indentation process presents a protuberance that grows on the front surface while the punch penetrates, that area has not been taken into account for the analysis because it corresponds to the dead material area generated under the punch (Figure 14).

The FEM study shows a strain concentration at the lower corners. This concentration should not be considered since it is influenced by the meshing. At the point of contact, the vertex would be infinite. In addition, a direct comparison with DIC cannot be made due to a low resolution. The low resolution is caused by the pattern and the resolution of the camera itself [28,29]. Though a direct comparison is not possible, another comparative analysis can be carried out like the one presented in this work.

Regarding the vertical displacements, the FEM model is stiffer than the workpiece. There is a vertical difference of the top of the samples when the DIC and FEM final images are compared. This difference could be explained by the fact that the 2D model is not accurate enough to reproduce the 3D part behaviour.

**Figure 15:**
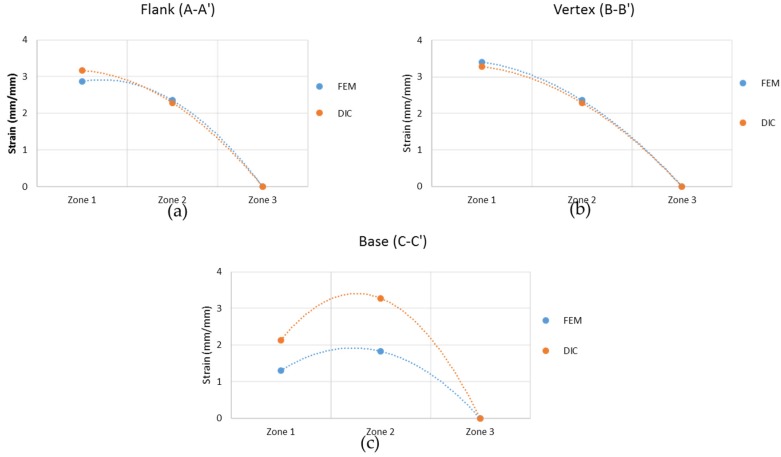
Von Mises strain values comparison between FEM and DIC focusing on the flank (**a**), the vertex (**b**), and the centre of the base (**c**).

The comparison between both methods, in order to validate the FEM model developed, must be centred in three different regions, represented with white lines on Figure 13a,b (regions A, B, and C for FEM and A’, B’, and C’ for DIC). Following these lines, three points are selected: Both extremes of the line (zone 1 and 3) and the middle point (zone 2). Figure 15 shows the comparison for the average strain values in those cases, presenting a good correlation between the results. For the area of the vertex of the punch, the difference is 7–11%. For the flank, there is a difference between 10% and 20%. Finally, for the middle line of the base, there is a higher disparity of 45%. This disparity on the base of the punch can be due to the dead material zone interference.

Both methods present the dead nose zone (Figure 13 and Figure 14). This zone is material that moves solidary with the punch and do not flow. This dead nose zone is produced in front of the punch in deep indentations, whether there is lubrication or not, and can affect the results obtained in that specific zone with the experimental method.

## 4. Conclusions

The main purpose of this work is to show that the developed FEM model allows for a correct simulation of the indentation process and can be used in future designs to evaluate the maximum forces needed in these kinds of processes and the final shape achieved before the procedure takes place.

To validate the FEM model, the DIC method was used as an experimental technique.

The material flow is analysed using FEM and DIC, showing the adequacy of each method. To guarantee a deep indentation avoiding cracking, tin (99% tin) workpieces were manufactured. Tin has characteristics of near to rigid-perfectly plastic behaviour, but it is also very sensitive to the strain rate. All the experimental tests were carried out at 5 mm/min, maintaining the same displacement rate and allowing for a wide frame capture for DIC.

A two-dimensional indentation setup was designed in FEM and DIC to study the deformation process. FEM and DIC analyses show an adequate correlation with the experimental results. The main findings that can be highlighted are:

- The stress-strain curve of the material tested, 99% tin, is experimentally obtained and satisfactorily introduced in the FEM code.
- The FEM punch force-displacement curve fits well with the experimental results.
- The non-contact technique, DIC, is an efficient method for the identification of the flow field and von Mises strains.
- The DIC and FEM Von Mises strain results show an adequate correlation taking into account the simplicity of the 2D models, presenting values with a difference between 10% to 20% (and 50% in the dead zone). It can be observed that the DIC results are always contained between the FEM results.

Notwithstanding, if precise FEM strain maps are to be obtained, a 3D model is needed. The real behaviour of the workpiece under the punch, and near the surface, cannot be studied as a two-dimensional problem. Indeed, in this zone it is necessary to take into account the three dimensional effects.

The disadvantage of FEM lies in the need to obtain the stress/strain curves of the materials being analysed. In study cases of materials like tin, that are highly influenced by strain rates, the stress/strain curve for each speed considered is needed. This is not necessary when working with materials like aluminium, as presented in previous works. This analysis confirms that DIC can provide full strain fields and can be used to examine manufacturing processes like indentation. Also, after the necessary image treatment, DIC offers the possibility to obtain different types of information such as damage, stress distribution, or the stress-strain curve.

## Figures and Tables

**Table 1:** Tin composition in %.

Sn	Sb	Cu	Other
99.95	0.02	0.002	0.028

**Table 2:** Mesh applied.

Number of Elements	10,000
Number of mesh windows	2 (relation 1/10)
Remesh maximum step increment	2

**Table 3:** General simulation settings.

Number of Simulation Steps	100
Step Increment to save	2
With equal die displacement	0.05

## Nomenclature

IFP: Incremental Forming Processes.
SBMF: Sheet-Bulk Metal Forming.
DIC: Digital Image Correlation.
FEM: Finite Element Method.
DAQ: Data Acquisition system.
C(*x, y, u, v*): Value of the correlation function for a given pixel in the position (*x, y*) that undergoes a horizontal (*u*) and vertical (*v*) displacement (reference image).
*n*: Subset size.
*I*(*x* + *i*, *y* + *j*): Value associated to the pixel in the position (*x* + *i*, *y* + *j*) (reference image).
Γ(*x* + *u* + *i*, *y* + *v* + *j*): Value associated to the pixel in the position (*x* + *u* + *i*, *y* + *v* + *j*) (deformed image).
(*x*, *y*): Desired point coordinates.(*u*, *v*): Displacement of the desired point.
*ε_xx_*: Longitudinal strains in x directions.
*ε_yy_*: Longitudinal strains in y directions.
*ε_xy_*: Angular strain.
*u_x_*, *u_y_*, *v_x_* and *v_y_*: Partial derivatives of the displacements (*u, v*).
